# Serum Level Alteration of IL-6, IL-1*β*, and IFN-*γ* in Groups of Healthy Adults with Oxidative DNA Damage in Najaf Governorate

**DOI:** 10.1155/2024/9048536

**Published:** 2024-09-02

**Authors:** Dhuha S. Saleh, Hayder S. Hussain, Hasan N. Al-Haidari, Samia K. Abbas, Ayaid K. Zgair, Seenaa M. Ali

**Affiliations:** ^1^ Department of Biology College of Science University of Baghdad, Baghdad, Iraq; ^2^ Department of Physics College of Science University of Baghdad, Baghdad, Iraq; ^3^ Department of Radiology King Hussien Medical Center Jordanian Royal Medical Services, Amman, Jordan; ^4^ General Directorate of Education in Najaf Ministry of Education, Dahuk, Iraq; ^5^ Laboratory Department College of Health and Medical Technology Sulaimani Polytechnic University, Sulaymaniyah, Iraq

## Abstract

**Background:**

Najaf governorate was recorded as one of the most polluted Iraqi governorates with increased cancer, autoimmune, and abortion cases. *Study Groups*. A total of 88 adult volunteers from three test groups were divided based on their inhabitance in different geographical regions in Najaf governorate. Group 1 (G1; *n*, 29) inhabitants of Al-Ansar, Al-Abbaseyeh, and Al-Manathera districts, Group 2 (G2; *n*, 27) inhabitants of 22 different scattered districts of the governorate, Group 3 (G3; *n*, 32) inhabitants of Kufa city and center districts in the old Najaf city. According to previous authors' findings, all participants had uranium contamination in their urine and blood samples, and also, they had DNA damage according to the level of urinary 8-OHdG compound. The control group 4 (G4; *n*, 25) were adult healthy Iraqi volunteers who were residents of the Sulaimaniyah governorate, which has low-level uranium pollution. The present study aims to determine the effect of uranium pollution and DNA damage on the immune system function in terms of estimating the levels of serum interleukin (IL)-6, interferon-gamma (IFN-*γ*), and IL-1 beta (*β*).

**Method:**

Enzyme-linked immunosorbent assay (ELISA) (Sandwich method technique) was used for estimating the serum cytokines levels in test and control groups.

**Results:**

A significant elevation of cytokines levels was reported as compared with the control groups (*p* ≤ 0.01). The level of IL-6 was 764.64 ± 24.12 pg/ml, 768.87 ± 19.64 pg/ml, and 735.62 ± 18.47 in G1, G2, and G3, respectively. The level of IFN-*γ* was 264.55 ± 19.17 pg/ml, 259 ± 18.76 pg/ml, and 261.20 ± 12.99 pg/ml for G1, G2, and G3, respectively. The level of IL-1*β* was 99.85 ± 10.81 pg/ml, 116.8 ± 10.71 pg/ml, and 83 ± 19.24 pg/ml in G1, G2, and G3, respectively. The levels of IL-6, IFN-*γ*, and IL-1*β* were 86.5 ± 22.9 pg/ml, 19.4 ± 2.8 pg/ml, and 16.1 ± 3.2 pg/ml in the sera of control (G4). The results showed significant statistical elevation with the corresponding *p* value cut-off *p* ≤ 0.01 in IL-6, IFN-*γ*, and IL-1*β* in the sera of three test groups as compared with the results of the control group.

**Conclusion:**

The change in the proinflammatory cytokines (IL-6, IFN-*γ*, and IL-1*β*) levels indicates a persistent inflammatory response in the participants and may reflect immune system impairment as a consequence of exposure to long-term low-dose ionizing radiation.

## 1. Introduction

The health consequences of environmental pollution are considered one of the most important research fields throughout the world. Environmental pollutants have adverse effects on human beings [1]. A large number of epidemiological studies reported the presence of a correlation between air pollution and increased morbidity and mortality in children. Vitamin D deficiency and impaired immune functions were recorded [2]. The living tissue damage caused by exposure to ionizing radiation results from the breakup of the molecules and the destruction of the biological processes *in vivo*. The cellular damage links linearly with the dosage received and depends on many critical variables including radiation type, intensity, energy deposited, and duration of exposure [3]. It has been reported that radiation causes ionization of atoms, molecules, cells, and tissues. It may affect some organs or the whole body [4]. The response of the immune system to irradiation depends on the dose and dose rate as well as on the radiation and the immune cell types [5]. The interaction between the immune system and ionizing radiation leads to the emergence of a new scientific field called radio-immunobiology [6].

The studies of the late effects of atomic bomb radiation on the immune system were started around twenty years after the Second World War. The most remarkable late effect of radiation is the functional and quantitative abnormalities of T and B cells in survivors exposed to dose ≥1.0 Gray [7], where Gray (Gy) is the unit of ionizing radiation dose measurement, and it has replaced the older “rad” designation, 1 Gy = 1 joule/kilogram = 100 rad, and it reflects the amount of energy absorbed in 1 kg of living body or matter. Gray can be used for any type of radiation (i.e., alpha, beta, neutron, and gamma), but it does not describe the biological effects of different radiations [8]. The immune system is affected by high-dose and low-dose radiation. The high-dose radiation effect has been demonstrated by epidemiological and experimental studies as suppression of immune function. High radiation doses > 2 Gy result in the massive killing of blood cells such as lymphocytes which are one of the important cells of immune cellular components in the immune system [9]. Low-dose radiation is both stimulatory and suppressing to the immune system [10–12]. The immune system in the human body is found to be affected by environmental pollution with uranium. Low levels of radiation could stimulate the immune system at least for a short period [13].

Interleukins (IL) as the most important mediators by which cells of the immune system communicate could be up- or down-regulated upon exposure to low-dose radiation [14]. In a study, it has been mentioned that negative health effects are known to occur in populations living near former and active mines, those effects may be associated with genotoxicity, the results of the study recorded that significant DNA damage was observed in peripheral blood samples from volunteers living near a deactivated uranium mine in Cunha Baixa village in Portugal, also a significant decrease of NK (Natural Killer) cells, and T-lymphocyte counts were observed in volunteers from the same village. DNA damage is one of the important causes of cancer cell development. NK and T-lymphocytes are the immune cells that play an important role in defense against cancer formation. The study suggested that the decreased level of those immune cells reflects impairment of the immune system of the population living beside deactivated uranium mines [15].

A study in Iraq/Baghdad on the increased cases of lung cancer recorded a significant decrease in NK both in patients and healthy control groups [16]. Ionizing radiation has been reported as one cause of oxidative stress and DNA damage [17]. Cellular water is a major target of ionizing radiation, ionizing radiation creates free electrons inside the cell, and because another molecule can easily pick up the free electrons, the resulting free radicals can affect dramatic and destructive changes in the cell and the intercellular fluid [18]. The excess production of reactive oxygen species (ROS) perturbs the balance of the cells to a more oxidant state and disturbs its normal biological function causing oxidative stress [19], and oxidative stress, resulting from ionizing radiation, will trigger a cascade of many events including alteration of the immune system [20]. Many experiments proved evidence that DNA and RNA are susceptible to oxidative damage. The DNA of mitochondria is 16 times more sensitive to radiation than nuclear DNA because it has no protection histone proteins like those within the cell nucleus; thus, the functionality of the cellular respiratory system (mitochondria) can be destroyed [18].

Proinflammatory cytokines play an important role in innate immune response and can be stimulated by bacterial lipopolysaccharide and proteins [21]. Proinflammatory cytokines such as interleukin IL-6, interferon-gamma (IFN-*γ*), and IL-1*β* are the major components of immediate early gene programs and as such can be rapidly activated after tissue irradiation [22]. IL-6 and IL-1*β* are secreted by macrophages, the first line of defense against inhaled foreign particles and one of the main cellular targets after the inhalation of depleted uranium [23]. IFN-*γ* is secreted by T- lymphocytes and NK cells which are the most sensitive immune cells to ionizing radiation exposure [24]. IFN-*γ* is one of the valuable indicators of blood lymphocyte activity after radiation exposure [25]. It is also involved in the periodic evaluation of the immune system before and after radiotherapy dose administration for cancer patients and periodic checkups of occupational radiation in nuclear countries [26]. The evaluation of IFN-*γ* was used to identify radiation effects on leucocytes since IFN-*γ* works as an activator for monocytes and macrophages [27, 28]. Those cytokines were chosen as immune parameters for the current study with consideration to abroad research plan which has been designed by the first author starting from the year 2010 and included the estimation of several cellular and humoral immune parameters needed for evaluation of the immune status of Iraqi people in Baghdad and other governorates [16, 29–31].

In the Najaf governorate, an increase in cancer cases, autoimmune diseases, and recurrent abortion cases were recorded in medical centers of the governorate during the last twenty years. This study aimed to determine to what extent, the uranium pollution and the DNA damage recorded in previous authors' studies affect immune system functions by estimating the cytokines serum levels of IL-6, IFN-*γ*, and, IL-1*β* in the three test groups based on their inhabitance districts in different geographical regions of Najaf governorate taken in consideration that all participants of G1, G2, and G3 had uranium pollution in their urine and blood samples [32, 33]. The fourth control group G4 in the present study included 25 volunteers of Arab Iraqi citizens residing in the Sulaimaniyah governorate which was known to have a low uranium pollution level [34].

## 2. Materials and Methods

### 2.1. Study Groups

Adult volunteers (*n*, 88), 67 males and 21 females, with age range less than 30 years (<30 years) to more than 40 years (>40 years), were divided into 3 test groups according to their inhabitance in different geographical regions in Najaf governorate. In previous authors' studies [32, 33], the *α*-radiation level was measured in terms of uranium concentration for all groups by using solid state nuclear track detectors (SSNTD) methodology with CR-39 detector, but there was no estimation for the *γ*-radiation level. Group one (G1; *n*, 29) inhabitants of Al-Ansar, Al-Abbaseyeh, and Al-Manathera districts near Al-Heera abandoned uranium mine which were known for more than 20 years as being environmentally polluted. The first affected area was the Al-Ansar neighborhood after a sudden outbreak of leukemia cases among children, and then increased cancer cases, recurrent abortions, and other diseases were recorded in the other two districts where the uranium concentration was 1.872 ± 0.06 *µ*g/L in blood serum and 1.838 ± 0.07 *µ*g/L in urine samples of G1. Group two (G2; *n*, 27) inhabitants of 22 different scattered districts in Najaf governorate. They were health workers with uranium concentration of 1.947 ± 0.05 *µ*g/L in blood serum and 2.021 ± 0.08 *µ*g/L in urine samples. In group three (G3; *n*, 32) inhabitants in Kufa city and center districts of old Najaf city, uranium concentration recorded was 1.804 ± 0.09 *µ*g/L in blood serum and 1.755 ± 0.08 *µ*g/L in urine samples. The test participants in those three groups are getting used to living in their regions, and they had no other choice. Some people may be exposed to more or less pollution than others. The degree of pollution for each region is not documented officially, but it was estimated on the base of certain disease or cancer cases outbreaks in certain regions within a certain period. The control group (G4; *n*, 25) was 18 females and 7 males with the age range of 18–58 years, healthy adult volunteers by medical examination, Iraqi Arab residents of Sulaimaniyah governorate, which is considered a low-level uranium polluted area [34]. Uranium concentration in this group (G4) was recorded as 1.474 ± 0.097 *µ*g/L in blood serum samples and 1.410 ± 0.11 *µ*g/L in urine samples. Previous findings of authors [32, 33] recorded DNA damage in terms of urinary concentration of 8-OHdG compound (biomarker) by using the enzyme-linked immunosorbent assay (ELISA) method. The levels of 8-OHdG compound were 49.81 ± 2.87, 47.72 ± 2.73, and 46.75 ± 2.52 ng/ml in G1, G2, and G3, respectively, while it was 4.7 ± 1.6 ng/ml in the control group (G4).

### 2.2. Blood Sample Collection

From May 2019 to August 2019, 88 samples (those divided into three groups) were collected. Before taking the blood samples, the adult volunteers were chosen as test participants based on the following criteria: sufficient activity, apparent look, no active disease, and no history of chronic diseases, and a volunteer who felt tired was excluded. Blood samples (3 ml) were sampled from the brachial vein, collected in a vacuum gel tube without anticoagulant, and left at room temperature for 10 minutes. The sera were obtained after centrifugation of blood at 3000 r.p.m. for 15 minutes and then transferred to microfuge tubes to store at −80°C till used [35].

### 2.3. Cytokine Estimation

ELISA (Sandwich method technique) was used to measure the human IL-6, IL-1*β*, and IFN-*γ*. The instruction of the manufacturing company (US Biological/USA) was followed to estimate the concentrations of the cytokines. Briefly, 100 *µ*l of serum and/or standard samples were placed in wells of microplate. The plate was covered with a plate sealer and incubated for 2 hr on a microplate shaker at room temperature. Each well was washed and aspirated three times and repeated for four washes using wash buffer, and complete removal of the liquid at each step is essential. 100 *µ*l of detecting cytokine antibody working solution was added to each well. The steps of incubation washing and aspiration were repeated. 100 *µ*l of substrate tetramethyl benzidine (TMB) solution was added to each well and incubated for 15–20 min. The plate was incubated at room temperature away from light, and then 100 *µ*l of stop solution (0.5 M HCl) was added to each well. The optical density was read for each well within 20 minutes using a microplate reader set at 450 nm. The concentration of the cytokine was calculated using the standard curve.

### 2.4. Statistical Analysis

The Statistical Analysis System (2012) program was used to detect the effect of different factors on study parameters. The least significant difference- LSD test (analysis of variation- ANOVA) was used to compare significance among the means of the study groups [36].

## 3. Results

**Figure 1:**
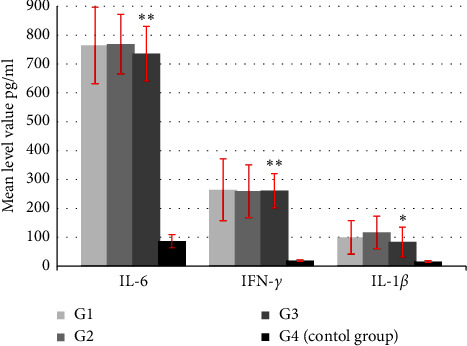
Comparison of IL-6, IFN–*γ*, and IL-1*β* between test groups G1, G2, and G3 and the control group (G4). The least significant difference- LSD (analysis of variation- ANOVA) was used for comparison of data. Data are expressed as the mean ± standard deviation (SD), and they differ significantly with the corresponding *p* value cutoff (*p* ≤ 0.01)^*∗*^, (*p* ≤ 0.01)^*∗∗*^.

The results of cytokines levels are illustrated in Table 1 and Figure 1. The values were presented in mean levels of the cytokines in the sera of all study groups.

### 3.1. Interleukin-6

The results of the present study recorded mean level values of IL-6 as 764.64 ± 24.12 pg/ml, 768.87 ± 19.64 pg/ml, and 735.62 ± 18.47 pg/ml in the test groups G1, G2, and G3, respectively, control group G4 recorded the mean level value of IL-6 as 86.5 ± 22.9 pg/ml, and there is a significant statistical difference (*p* ≤ 0.01) when comparing the results of the three test groups with those of the control group.

### 3.2. IFN-*γ*

Interferon-*γ* mean level values were recorded in the present study as 264.55 ± 19.17 pg/ml, 259.51 ± 18.76 pg/ml, and 261.20 ± 12.99 pg/ml in the test groups G1, G2, and G3 respectively, while control group G4 recorded the mean level value of IFN-*γ* as 19.4 ± 2.8 pg/ml, and there is a significant statistical difference (*p* ≤ 0.01) when comparing the results of the three test groups with that of the control group.

### 3.3. Interleukin-1*β*

The mean level values of serum IL-1*β* in this study were recorded as 99.85 ± 10.81pg/ml, 116.80 ± 10.71 pg/ml, and 83.86 ± 9.24 pg/ml in the test groups G1, G2, and G3 respectively, control group G4 recorded 16.1 ± 3.2 pg/ml for IL-1*β* mean level value, and there is a significant statistical difference (*p* ≤ 0.01) when comparing the results of the three test groups with that of the control group.

## 4. Discussion

The results of the current study recorded elevation in the levels of the cytokines IL-6, IFN-*γ*, and IL-1*β* in all participants of the three test groups G1, G2, and G3 by comparing the mean level values of IL-6, IFN- *γ*, and IL-1*β* of the test groups with those of the control group, and a significant statistical difference was recorded with a corresponding *p* value cut-off *p* < 0.01 (Table 1, Figure 1). By those results, the immune response of the participants was studied through measurements of serum cytokines. The previous finding of the authors recorded uranium contamination and DNA damage in all participants of the current study [32, 33]. Some districts like Al-Ansar had a history of leukemia outbreaks in children in the 1990s as recorded by the Ministry of Health (MoH) in Iraq. Elevated levels of human exposure to ionizing radiation due to naturally occurring radionuclides and/or human activities are known as naturally occurring radioactive material with the abbreviation of NORM [37]. Recently, it has been recorded according to Iraq's national cancer registry during 2017-2018, and there were more than 31500 cancer patients recorded in Iraq. The mortality rate of cancer is around 11% [38]. Additionally, Iraqi hospitals recorded about 1674 patients with leukemia, 791 patients with gastrointestinal cancer, and 2123 patients with lung cancer every year [39].

The cytokines elevated levels in control healthy Iraqi people were noticed since the year 2010 when broad plan studies were started. IL-6 mean level value was recorded as (21 ± 8.5 pg/ml) in the healthy control group in a study of increased respiratory disease incidence in Baghdad [29]. Another local study in Baghdad on rheumatoid arthritis patients recorded the IL-1*β* level in the healthy control group as (52 ± 27 pg/ml) [40]. IL-2 was also estimated in Iraqi women with recurrent abortions, and the level of this cytokine in the sera of control healthy nonpregnant women was 36.48 ± 13.19 pg/ml [31]. Normal levels of those cytokines are usually lie in narrow low ranges in all humans throughout the world and sometimes undetectable, and this fact was confirmed in many studies of other countries like Tunisia [41], Saudi Arabia [42], and Turkey [43]. Cytokine serum level elevation was recorded in mines workers in different countries, and highly elevated IL-6 levels have been recorded with phosphate miners in Tunisia where it reached 321 pg/ml [41]. In China, it was found that IFN-*γ* and IL-6 were upregulated in uranium miners who worked more than 5 years underground and they produced persistent inflammatory responses when they received more than 20 mSv per year [10].

An *in vitro* study in China's Nuclear information center recorded that the uranium mineral dust stimulates alveolar macrophages to release higher concentrations of cytokines, i.e., IL-6 (1335 U/ml) [44]. IFN-*γ* and IL-l*β* had significantly higher concentrations in mercury-exposed gold miners [45], and also, IL-l*β* was found to be upregulated directly by irradiation effect. IL-l*β* is known to be a factor that regulates other inflammatory molecules, and it has been recorded as a critical factor in the production of skin fibrosis which results as a consequence of radiation therapy [46]. Cytokine may also be elevated under the effect of natural ionizing radiation where IL-2 was reported as 760 ± 275 pg/ml in healthy control individuals residing in an Iranian village with the normal level of natural ionizing radiation [11]. It was recorded that the natural radiation effect on living cells depends on the radiation exposure dose [47]. In our present study, some of the participants are inhabitants of the Al-Manathera district near the Al-Heera abandoned uranium mine, and they have blood contamination with uranium, DNA damage [32, 33], and also elevated levels of the cytokines tested. In the present study, these findings are in agreement in some way with those of a study in Portugal on a human population residing in a village near a deactivated uranium mine where these residents recorded blood contamination with uranium, significant DNA damage, and a significant decrease in natural killer cells and T lymphocytes, and results suggested that inhabitants near the deactivated mine may have a higher risk of serious diseases like cancer [15].

Ionizing radiation at low doses for a long-term effect is considered a cause of cancer induction in humans [48]. Although the health effects are known to occur in populations living near former and active mines, there are relatively few studies focusing on it [49]. Uranium contamination may come from inhalation of dust or through ingestion of contaminated food or water [50, 51]. In the present study, some participants are living near the abandoned uranium mine of Al-Heera, but other participants reside in a region far away from that of mine, a fact which suggests the presence of another source/sources of low-dose uranium irradiation. High-dose ionizing radiation exposure affects the immune system by suppressing its functions, while the low-dose radiation effect is both stimulatory and suppressing of the immune system [7, 52]. It has been recorded that low doses of ionizing radiation exposure may cause a permanent alteration of immune fitness causing a wide spectrum of pathophysiological events, and also, low-dose effects may be silent until late after exposure [53]. IL-6, IFN-*γ*, and IL-1*β* are proinflammatory cytokines, and they can be activated rapidly after tissue irradiation. The radiation danger to the body is to cause an inflammatory response in the exposed site, and infiltration of cells, primarily neutrophils, was followed by lymphocytes and macrophage infiltration [54]. IL-1*β* is a highly effective proinflammatory mediator at the tissue level which results in vasodilation and attraction of granulocytes, and IL-1*β* could join innate and adaptive immunity because of its nature as a lymphocyte activator factor [40, 55]. IL-1*β* is one member of the IL-1 family, and it has been reported that IL-l can serve as a signal that initiates radioprotective events *in vitro* [56]. IFN-*γ* and IL-6 coordinate the inflammatory response [10]. The studies at present time focused on the biological effects resulting from low-dose exposure where the end point of pathophysiological mechanisms is not due to the direct effect killing of the cells by ionizing radiation but most probably immune-mediated [6].

In our present study, the increased recorded levels of proinflammatory cytokines are in agreement with those of atomic bomb survivors more than 50 years after radiation exposure in which IFN-*γ*, IL-6, IL-10, and TNF-*α* increased expression providing evidence of persistent inflammatory response [57]. It has been reported in a study that IL-6 is a procarcinogen inflammatory cytokine that plays an important role in the development of lung cancer in uranium miners [58]. It was mentioned in a previous study that continuous dysregulation of IL-6 will lead to cancer formation and different inflammatory and autoimmune diseases' development [59]. Exposure to environmental uranium has been linked with several health consequences and can exert toxic effects on several important physiological processes including kidney functions, bone development, and hematopoiesis [49]. Ionizing radiation (low-dose DU), heavy metal, sand, and other toxic chemicals destroy the functionality of the cellular respiratory system (the mitochondria), disrupt the chemistry of enzymes and hormones, frustrate the normal cellular detoxification and repair mechanism, and leave a person alive but chronically ill [18]. Referring to the above, the effects of prolonged, low-dose radiation exposure may explain the mechanism by which healthy individuals transformed gradually into chronically diseased populations. The elevation of cytokines levels in the current study is a clear sign of immune function impairment since the oxidative stress resulting from ionizing radiation exposure is known to be a factor that triggers a cascade of many events including function alteration of the immune system [20]. According to our results, we suggest that the inhabitants of the mentioned districts in Najaf governorate have a higher risk of developing serious diseases like cancer, and this suggestion is in agreement with that of similar studies concerning the negative effect of uranium pollution on the immune system [15, 53], and further studies with a larger sample size may result in a significant progress concerning understanding the effect of chronic low-dose radiation on immune function in Iraqi people.

## 5. Conclusion

Elevated levels of the proinflammatory cytokines (IL-6, IFN-*γ*, and IL-1*β*) in the test groups G1, G2, and G3 as compared with results of the control healthy group G4 may indicate persistent inflammatory response in those participants which may reflect impairment in the immune system functions as a result of cumulative long-term low-dose exposure of ionizing radiation, and these findings may be one cause of increased cases of high disease incidence in Najaf governorate.

## Figures and Tables

**Table 1:** The levels of IL-6, IFN-*γ*, and IL-1*β* in the sera of human test groups (G1, G2, and G3) and control group (G4).

Biomarkers		G1	G2	G3	G4 control group	*p* value	LSD value
IL-6 (pg/ml)	Range	614.643–1093.214	643.214–1036.071	614.643–971.786	34.3–128.2	0.0009	71.7^∗∗^
	Mean ± SD	764.6 ± 132.5	768.8 ± 102.7	735.6 ± 94.3	86.5 ± 22.9		

INF-*γ* (pg/ml)	Range	99.22–454.78	27.00–415.89	77.00–432.56	15.8–27.7	0.0001	57.8^∗∗^
	Mean ± SD	264.5 ± 106.9	259.5 ± 91.1	261.2 ± 59.7	19.4 ± 2.8		

IL-1*β* (pg/ml)	Range	6.47–238.05	9.11–219.63	−14.58–159.11	11.4–24.7	0.008	48.4^∗^
	Mean ± SD	99.8 ± 57.7	116.8 ± 56.2	83.9 ± 51.8	16.1 ± 3.2		

The least significant difference (LSD) (analysis of variation, ANOVA) was used for comparison of data. Data are expressed as mean ± standard deviation (SD), they differ significantly with the corresponding *p* value cutoff (*p* ≤ 0.01)^*∗*^, (*p* ≤ 0.01)^*∗∗*^.

## Data Availability

The data that support the findings of this study are available on request from the corresponding author.
